# Can’t Contracting Be Relational? Comment on "Alignment in the Hospital-Physician Relationship: A Qualitative Multiple Case Study of Medical Specialist Enterprises in the Netherlands"

**DOI:** 10.34172/ijhpm.8651

**Published:** 2024-10-22

**Authors:** David Hughes

**Affiliations:** Faculty of Medicine, Health and Life Science, Swansea University, Swansea, UK.

**Keywords:** Netherlands, Hospitals, Medical Specialist Enterprises, Physician Reimbursement, Relational Contracting, Organisational Governance

## Abstract

Ubels and van Raaij provide a valuable account of the operation of novel hospital/medical specialist enterprise (MSE) contracts in a Dutch healthcare system shaped by market reform. However, their analytical distinction between the separate domains of contractual and relational governance frames the contractual domain more narrowly than does the relational contract theory widely deployed in socio-legal studies. The authors’ conclusion that contract plays little or no part in governing relations between hospitals and MSEs leads them to underplay a wider realm of contractual practices that develop in the shadow of the written contract. Apparent non-use of contracts in favour of shared planning, compromise and extra-legal solutions only takes the form it does because of the potential application of the available legal framework. Larger qualitative field studies involving a more extensive combination of interviews and observations may be needed to gain fuller insights into the relational dimensions of the contracting process.

 Ubels and van Raaij^1^ have published a very useful study of a novel form of provider/provider subcontracting in the Dutch healthcare system, and my comment is intended less as a criticism of their approach than a reflection on future directions for theoretical development. In particular, I argue that the analytical split between contractual and relational governance associated with the law and economics tradition, may be less productive in this context than the concept of relational contracting^2^ developed in socio-legal studies.

 The Dutch study is noteworthy for its empirical focus, particularly its direct engagement with actors involved in managing an innovative form of contracting between hospitals and their self-employed physicians. The Netherlands is an interesting outlier among Western European social health insurance systems, because following reforms that introduced regulated competition between private insurers it moved further towards market organisation than its neighbours. Over time the policy framework was adjusted to enable health insurers to move towards active, value-based purchasing from contracted hospitals. Initially, funding was channelled through separate agreements with hospitals and self-employed physicians with admitting rights. Ubels and van Raaij’s paper breaks new ground because it examines the policy change that occurred in 2015 when the Dutch government required all reimbursement claims to insurers to come from hospitals, which led self-employed doctors to respond by creating medical specialist enterprises (MSEs) as legal entities that could contract with hospitals on their behalf regarding physician payments. The study examines these contracts, and how insurers, hospitals, MSEs, and professionals are implicated in a complex set of principal/agent relationships. The parties negotiate to allocate risks and benefits so as to maximise advantage, but in a context where management must also take account of professional power and the maintenance of harmonious relations and trust within hospitals.

 My quibble with the paper concerns the chosen theoretical framework, and what I take to be the omission of important parts of the story about what we might term the contractual domain. Among the main conclusions of the Dutch research are: (*a*) that the study hospitals veer more towards relational governance than contractual governance, (*b*) that the relative inattention to the contractual documents runs counter to findings for business contracting, and (*c*) that “the contract is clearly not used as such [to govern the relationship] in the healthcare setting” (p. 10). This last finding regarding the non-relevance of contractual analysis is the main point I want to challenge, and I will instead argue for an expanded understanding of contractual governance that covers a wider range of behaviours and practices enacted in the shadow of the formal contract.

 Part of the problem is that the law and economics literature favours a narrower conception of the contractual domain than is found in socio-legal studies scholarship. From the economics side, Zenger et al^3^ suggest that contracts are a formal governance mechanism incorporating rules that “are readily observable through written documents or rules that are determined and executed through formal position, such as authority or ownership” (p. 277). This concern with formal contractual documents resembles the tendency in classical and neo-classical contract scholarship to fixate on the written word of the contract and established legal doctrine, rather than the context of the agreement and facts of the case. Yet much of the empirical research on contracts since the 1980s, especially work in the “law-in-action” tradition, has rejected the classical approach and turned more towards relational contract theory.^4^ Although divergent approaches such as the new institutionalism^5^ and neo-formalism^6^ have gained influence, the relational contracting literature remains an important reference point for researchers wishing to examine contracting practice in institutional or market contexts,^7,8^ and in my view has high potential relevance to the Dutch hospital case.

 Conspicuous by its absence from Ubels and van Raaij’s paper is the work of Ian Macneil, whose seminal writings delineated the main elements of relational contract theory.^9^ Macneil questioned how far classical contract theory with its emphasis on the planning and enforcement of discrete, fully “presentiated” contracts (contracts that bring the future into the present by making provision for the full range of contingencies that might affect performance) corresponded with real-world contracting practice.^10^ He noted that most business contracts are not designed to cover discrete, one-off transactions, but rather are renewable agreements intended to cover a series of ongoing transactions between parties known to each other. Entering a contract is not simply about making available legal remedies for non-performance, but a means of shaping the exchange relationship. Macneil argued that contracting behaviour, if not the form of the written contract, generally reflects the value placed upon this ongoing relationship. This ranges across multiple activities, including contract planning, negotiation, document drafting, performance monitoring and review, negotiated variations, handling disputes and dispute resolution.

 Especially where a long-term continuing relationship is involved, the written contract may be sparse. It will usually be impractical or impossible to anticipate all contingences in a fully presentiated or “complete” contract, so that a degree of tolerated flexibility is generally present. Macneil argues that contracts lie across a spectrum, ranging from discrete contracts suited to one-off transactions in low-trust situations to the relational contracts characteristic of longer-term relationships. At the “relational” end, less attention is given to presentiation, and agreements are designed with sufficient flexibility to allow future determination of relevant matters and facilitate contract variations, as well as typically including clauses mentioning shared goals, reciprocal indemnification rights and dispute resolution arrangements that would minimise damage to the ongoing relationship.^11^ The Dutch hospital-MSE contracts have some of these characteristics, as indeed is evident from the relational features Ubels and Raaij list in Figure 1 and Table 2, such as mediation or arbitration, normative paragraphs, and multi-year contract terms.

 Contracts in other domains frequently include both discrete and relational elements, with many theorists arguing that both components are always present to some degree. Macneil explains that even within a generally relational contract some issues may still be best managed using relatively discrete contract law, so that relationality does not mean disapplying legal contract norms. He writes that: “The need for a contract law system enhancing discreteness and presentiation will never disappear … such a system will, however, continue to rub in an unnecessarily abrasive manner against the realities of coexistence with relational needs for flexibility and change. Only when the parts of the contract law system implementing discreteness and presentiation are perceived … not as an independent system but only as integral parts of much larger systems, will unnecessary abrasion disappear” (p. 888).^12^ The larger system centres on the norms of the exchange relationship, and in relational contract theory this is seen as an aspect of contractual governance, rather than a separate relational domain.

 Macneil’s work suggests that inattention to written contracts does not necessarily mean that behaviour falls outside the domain of contractual governance, and there are hints that this may well be the case in the Dutch hospitals, as when we are told that hospital-MSE contracts “were rarely consulted in any hospital, both in cases where contractual as well as relational governance was observed” (p. 7), and that “contracts were unexpectedly found to play no important role in the subsequent relationship, even in the cases that relied more on contractual governance” (p. 10).

 Does this mean that activity in the Dutch hospitals crossed the line from the contractual domain to a relational domain? It might be argued that certain relational contract theorists themselves describe a non-contractual domain. Macaulay’s classic paper on non-contractual relations in business,^13^ based on interviews with businesses and their legal advisers, is often interpreted in this way. Macauley found that more attention was given to custom and other informal social practices than to the clauses of the contract. Indeed written contractual terms were often incomplete, particularly in respect of performance requirements. Rather than relying on contractual remedies to ensure satisfactory performance, businesses oriented more to considerations such as maintaining a good relationship and preserving reputation. Some respondents suggested that too much detail in contracts might have the negative consequence of leading the other party to perform according to the letter rather than the spirit of the contract, and also because detailed performance clauses might indicate a lack of trust that raised doubts about the suitability of the contracting partner. Contrary to Ubels and van Raaij suggestion, many empirical studies undertaken since Macauley’s study report that private businesses often conduct relations with their contracting partners with minimal reference to the written contract.

 However, Campbell has argued that apparent non-use is more a turning away from exclusive reliance on classical contract norms than non-use of contractual governance in the wider sense.^14^ He explains that Macauley did not find that legal rules ceased to have force, but rather that businesses made pragmatic use of non-legal norms to com­plement legal ones. Macauley, like Macneil, insisted that to “understand the functions of contract the whole system of conducting exchanges must be explored more fully” (p. 163).^15^ Campbell writes that as long as parties need to respond flexibly to contingent events “contractual practice must be beset by a schizo­phrenia between the classical law’s promise of performance of primary obligations and the lesson of actual complex contracting, which is that, if it is to be welfare-enhancing, it has to be a matter of compromise and settlement; in essence, co-operation” (p. 172).^14^ However, co-operation is not about altruism, but rather a mutual recognition that each party should obtain economic benefit from the continuing relationship. If this is absent then the possibility of holding a partner to the terms of the contract remains. What emerges is a complex interplay between the use of legal and non-legal remedies dependent of the benefits and disbenefits of using contract to solve exchange problems in given situations. Campbell raises the question: why if apparent non-use works so well do businesses still enter contracts? He argues that the answer is that the contracting process both allows parties to plan the form of their exchange relationship and provides security for the exchange if problems emerge. Put differently, parties may leave the written contract unread in a drawer, but in doing so they do not escape the framework of law of which the contract is a part. They may calculate that informal remedies are less costly and more convenient, but the legal constitution of the relationship as a contractual relationship remains in force if things go wrong.

 With the above in mind, Ubels and van Raaij’s empirical findings suggest that hospital-MSE contracts contain the mix of discrete and relational elements predicted by relational contract theory, with several appearing to lie towards the relational end of the spectrum. Moreover, it seems clear that the organisational behaviour that surrounds the written contracts – what we might call the wider contracting process – would have been different had not a written contract existed. In my view the authors could therefore have presented their findings in a way that reaffirmed the relational dimension of contracting, without needing to argue that, in contrast to the situation in the business world, the contract does not govern relationships in Dutch healthcare settings.

 What would a situation in which contractual governance played no part look like? Could the parties arrive at a form of private ordering that allowed them to disregard the potential application of contract law? Is that what the relationship between hospitals and MSEs might become? On close examination there seem few real-life examples in market economies where an extra-legal order of this kind emerges, except perhaps an order is imposed through hierarchy rooted in economic dependency.^15^ The regulated market within which Dutch hospitals operate rests on a strong statutory foundation that limits the possibility for either a powerful hospital administration or dominant medical profession to enforce a private order that favours one side. Another possibility, were all hospital doctors to be made salaried employees, is that physician remuneration and terms of service become largely internal organisational matters based on standard employment contracts and ongoing negotiations, but with the primary contractual instrument that determines resource flows being the agreement between insurer and hospital. Again such a radical shift seems unlikely in the Dutch context.

 As Ubels and van Raaji say, one of the strengths of their study is its use of the qualitative method, but it might be observed that serious field research demands a more intensive effort. A pair of interviews in each of five study hospitals (10 interviews) is probably insufficient to understand the wider process of contract planning, negotiation, performance monitoring, dispute resolution, and so on that contractual governance entails. Several past UK studies of purchaser/provider contracting, like the example cited here,^16^ have involved in-depth fieldwork over one or more annual cycles, including attendance and audio recording of contract negotiation and monitoring meetings, as well as much larger interview samples. Future Dutch work could usefully extend this useful preliminary study by undertaking more extensive fieldwork-based investigations.

## Ethical issues

 Not applicable.

## Conflicts of interest

 Author declares that he has no conflicts of interest.
